# What Do Older Adults Consider a Good Death?

**DOI:** 10.1093/geroni/igaa057.1357

**Published:** 2020-12-16

**Authors:** Ellen Csikai, Quentin Maynard

**Affiliations:** 1 University of Alabama, Tuscaloosa, Alabama, United States; 2 University of Southern Indiana, Newburgh, Indiana, United States

## Abstract

Without improved understanding and communication about what constitutes a ‘good death’, an extended dying process that does not attend to how serious illness affects older adults’ quality of life near the end of life is likely (IOM, 2014). The primary aim of this study was to understand the concept of a ‘good death’ as defined by older adults. A scoping literature review of qualitative and quantitative research studies (published in peer-review journals since 2000) involving only community-dwelling older adults was conducted, using key search words: good death, dying well, older adults/elderly/seniors, gerontology/geriatrics and Boolean operators in Abstracts in Social Gerontology, CINAHL Plus, PubMed, Academic Search Premier, PsychINFO. A total of 344 articles met the search criteria. Following initial systematic screening of titles/abstracts and full text review, five articles remained that met inclusionary criteria. Therefore, this analysis is based on a total of five articles. Each study was qualitative, with small samples and diverse populations of older adults. Among the factors perceived important, studies’ shared results were dying in sleep/natural death/peaceful; family connection (e.g. time for closure, not being a burden) and faith/religiousness (e.g. getting ‘right’ with God, living with faith). Conceptions of a ‘good death’ often reflect primarily psychosocial/quality of life aspects, in addition to medical concerns. Health professionals need to know how a good death is defined by each individual in order to ensure holistic care and quality of life at the end of life according to older adults’ expressed wishes.

